# Consistency Index-Based Sensor Fault Detection System for Nuclear Power Plant Emergency Situations Using an LSTM Network

**DOI:** 10.3390/s20061651

**Published:** 2020-03-16

**Authors:** Jeonghun Choi, Seung Jun Lee

**Affiliations:** Ulsan National Institute of Science and Technology, 50 UNIST-gil, Ulju-gun, Ulsan 44919, Korea; jhchoi@unist.ac.kr

**Keywords:** sensor fault detection, consistency index, machine learning, emergency situations, misdiagnosis prevention

## Abstract

A nuclear power plant (NPP) consists of an enormous number of components with complex interconnections. Various techniques to detect sensor errors have been developed to monitor the state of the sensors during normal NPP operation, but not for emergency situations. In an emergency situation with a reactor trip, all the plant parameters undergo drastic changes following the sudden decrease in core reactivity. In this paper, a machine learning model adopting a consistency index is suggested for sensor error detection during NPP emergency situations. The proposed consistency index refers to the soundness of the sensors based on their measurement accuracy. The application of consistency index labeling makes it possible to detect sensor error immediately and specify the particular sensor where the error occurred. From a compact nuclear simulator, selected plant parameters were extracted during typical emergency situations, and artificial sensor errors were injected into the raw data. The trained system successfully generated output that gave both sensor error states and error-free states.

## 1. Introduction

The state awareness of complex systems such as aircraft systems, oil and gas facilities, and nuclear power plants (NPPs) comes from a myriad of sensors installed at desired locations. The sensors receive external stimuli from connected components and transport the signals to system users or other related systems. In an NPP, over 10,000 sensors and detectors are equipped to monitor the condition of all components [1]. To maintain the safety and stability of an NPP operation, instrumentation and control systems are employed that collect plant parameters from the sensors and process the data. These processed data are then presented to the operators in the main control room via measurement values or alarms. The operators monitor the plant state and cope with any abnormal situation depending on the given information [2]. In this flow of information, faults in the sensors can lead to operator misunderstanding, which could result in critical human error.

The Three Mile Island (TMI-2) accident, a severe accident that caused a partial meltdown of an NPP reactor core, is an example of how sensor error can contribute to exacerbating accident consequences. The TMI-2 accident started from a mechanical malfunction in feedwater pumps; during the accident sequence, one indicator showed the wrong information to the field operator, which led to a critical human error that worsened the accident [3]. Such a consequential example makes it clear that errors in sensors or indicators can have significant effects on accident situations.

In the nuclear field, online monitoring (OLM) techniques have been developed to be the representative faulty sensor detection method. However, these techniques are inappropriate to cover NPP emergency situations that include complex and nonlinear changes of plant parameters because they have been developed to extend the calibration period in steady operations only [4,5,6]. To overcome this limitation of existing methods, this paper proposes a novel consistency index and applies it to a machine learning model. The consistency index is a measure to quantify deviations of sensor values from the real values. The machine learning model is first trained with error and error-free data with consistency index labels. Then, the trained model produces a consistency output by taking typical emergency situation data as input, and then classifies the state of the sensors by the established logic. The suggested sensor fault detection system enables the monitoring of all parameters included as inputs of the system. It is believed that this system could prevent certain types of critical human error such as resulting from sensor error, and could also contribute to a practical background for data-driven methods or operator support systems based on plant parameters in NPPs [7].

## 2. Related Research and Research Purpose

### 2.1. Sensor Fault Detection and Identification

Sensor failure events can occur in diverse ways because there are various types of sensor networks [8]. Sensor fault detection is classified by the existence of redundant sensors that measure the same process parameters over a similar operating range. For physically redundant sensors, hardware redundancy approaches are applied such that sensor errors can be detected from simple mathematical models, for example, averaging. If physically redundant sensors are not installed, analytical redundancy approaches are applied that estimate sensor error from the relation between a range of different target sensors. These approaches can be classified into model-based [9,10], knowledge-based [11,12,13], and data-driven methods [14,15,16,17] depending on the understanding of the depth of the system and the available amount of data. Jiang et al. (2011) demonstrated this classification of general sensor fault detection [18]. In recent industrial research, fault detection and monitoring methods for industrial cyber-physical systems have been suggested using novel principle component regression methods and other data-driven approaches [19,20]. Additionally, statistical pattern recognition methods have been applied to the monitoring and fault detection of industrial bearings [21,22]. 

### 2.2. Online Monitoring (OLM) Techniques and the Proposed Model

In 1995, a US Nuclear Regulatory Commission (NRC) report concluded that OLM techniques are able to monitor field sensors and signals [23]. These techniques are designed to monitor the performance of instrument channels so as to extend the calibration intervals required by technical specifications. Instrument conditions are tracked by OLM via the application of hardware redundancy approaches and data-driven methods. Applications of the developed OLM techniques include the auto-associative neural network [24], nonlinear partial least squares [25], the multivariate state estimation technique [26], and auto-associative kernel regression [27]. In more recent research, principle component analysis, deep belief networks [28], and singular vector decomposition [29] have been adopted for OLM. Previous data-driven methods only considered the steady-state condition with small uncertainty in the calibration of target sensors [4,5,6]. Their general process was to reconstruct sensor signals and perform a residual analysis in which the estimated signals were compared to measured parameters [30].

Methods that reconstruct sensor signals and calculate the residuals from the measurements have advantages in both error detection and sensor value estimation under normal operating conditions; however, these methods are not suitable for emergency situations in nuclear plants because a reactor trip and subsequent actuation of safety systems result in highly unstable plant parameters. In this context, this research aims to construct a framework for detecting faulty sensors during NPP emergency situations. Using a deep neural network with a consistency index, the model monitors for sensor error during rapid changes of all plant parameters following a reactor trip by considering multivariate inputs, or in other words, a large number of sensor conditions. The model is trained with numerous time series data, namely the uncertain and complex variables of the early reactor trip phase. The sensor error detection model, in this work, aims to monitor the soundness of target sensors as expressed by a consistency index of the sensors. While the system does not estimate plant parameters, it directly displays the current state of the sensors during NPP emergency situations. The output mainly focuses on whether or not the measured sensor values deviate from the real values.

## 3. Operator Actions in Emergency Situations

To ensure NPP safety, reactor trips are initiated in response to the detection of plant parameters deviating outside of predetermined set points. Control rods are immediately inserted into the reactor, leading to a rapid decrease of reactivity. This is connected to a decrease in reactor thermal power and rapid changes in all plant components [31], for example, turbine trips, valve openings and closings, and the actuation of safety systems, among many others. The irregular situations that cause reactor trips are called emergency situations in the nuclear field. After the indication of a reactor trip, operators in the main control room perform emergency operating procedures to mitigate the accident consequences that caused the plant parameters to exceed the reactor protection system set points or engineered safety feature set points, or other established limits [32]. According to the relevant emergency operating procedure, operators cope with the symptoms of the early trip phase and diagnose the accident.

Here, accident diagnosis depends on the process parameter values or their trends [33]. In terms of the time required for accident diagnosis, a safety report published by the International Atomic Energy Agency recommends that operators complete the diagnosis of an accident within 15 min after the first indication of the accident [34]. From the diagnosis results, operators select the appropriate optimal response procedure (ORP), which contains the tasks required to mitigate the specific accident based on the current symptoms. Typical ORPs cover a reactor trip, loss of coolant accident (LOCA), steam generator tube rupture (SGTR), excess steam demand event (ESDE), loss of all feedwater (LOAF), loss of forced circulation, loss of off-site power, and station blackout [35]. 

Because each ORP includes contextual tasks, a misdiagnosis of the accident could result in a critical omission of the appropriate mitigation action or an error of commission. Considering the lesson from the TMI-2 accident, wrong displays of plant parameters during an accident sequence bring a high risk of human error, which will result in even bigger consequences following the diagnosis steps. In some specific circumstances, even a single sensor parameter can be a critical factor to diagnose the accident, for example, an increase in secondary system radiation is a strong indicator of SGTR. Therefore, an error in such a critical sensor during an emergency situation can possibly lead to misdiagnosis. Lee et al. (2009) showed that indicator failure is one of the factors influencing misdiagnosis error [36].

## 4. Method

**Figure 1:**
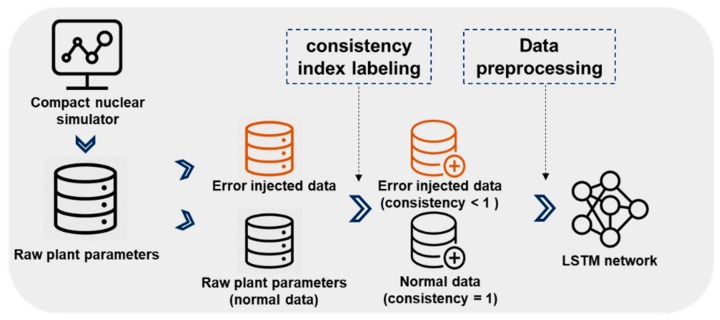
Data processing overview for sensor error detection.

An overview of the data processing involved in the developed system is shown in Figure 1. Raw data matrices from a compact nuclear simulator (CNS) are processed by injecting errors, and the consistency index is labeled on every time series sensor parameter. Then, the labeled data are used to train the machine learning model, here a long short-term memory (LSTM) network. The trained model generates consistency values for every sensor in response to the test data. The following subsections describe the related elements in detail.

### 4.1. Consistency Index

In this study, a consistency index that expresses the soundness of measurements is suggested. At first, the consistency index has values of 0 or 1, representing faulty and sound sensors, respectively. But such a binary classification of sensor states can result in a lowering of the threshold that judges sensor failure, because machine learning models can easily regard a normal sensor (with no error) as a faulty sensor in response to small deviations from, for example, sensor oscillation [37] or untrained sensor values. Therefore, a separate evaluation of sensor measurements is needed to avoid false detection of sensor error for measurements near the real value. In this case, the consistency index value is measured based on relative measurement accuracy; within about a 10% error, the consistency index is calculated from the square of the relative measurement accuracy. It has previously been shown that relative measurement error or accuracy can be used to quantify the performance of instrumentation or the quality of data [38]. Equations (1) and (2) give the relative measurement error (ε) and the consistency index (C), respectively, as:

(1) $$\varepsilon_{}=\frac{{\tilde{A}}_{}-A_{}}{A_{}},$$

(2) $$C_{i,t}=\left\{\begin{array}{c} {\left(1-\varepsilon_{i,t}\right)}^{2}={\left(1-\left|\frac{{\tilde{A}}_{i,t}-A_{i,t}}{A_{i,t}}\right|\right)}^{2}\;,\;\;\;0\leq \varepsilon_{i,t}\leq 0.1\\ 0\;\;\;\;\;\;\;\;\;\;\;\;\;\;\;\;,\;\;\;\;\;\;\;\;\;\;\;\;\varepsilon_{i,t}>0.1 \end{array}\right.,$$

where Ã and A are the measured and real values, respectively, i is the parameter number, and t is time. As seen in Equation (2), the consistency index C derives from the relative measurement error ε.

The consistency index is labeled on every time series parameter. All normal data and sensor error-injected data during the emergency situation are processed as inputs, with the trained model generating consistency output from the sensor parameter inputs. Without accident information, the model estimates the sensor states considering the analytical relation between other sensors and the temporal relation between early reactor trip sequences. Normal sensor trends are trained with the normal data, and parameter deviations are trained with the error-injected data. The system directly lowers the consistency index for anticipated sensor errors and accidents, and high computing power makes it possible to use a large number of parameter inputs and train for complex changes. Consequently, the consistency index value output of the suggested model replaces parameter reconstruction and residual analysis with a simpler process.

### 4.2. Sensor Error Modes

Sensor failures or uncertainties can occur from external or internal environmental causes. In previous applications of OLM techniques, typical sources of uncertainty were considered to establish the allowance of channel uncertainties. Examples of these sources include temperature and pressure (external), and systematic structure and sensor type (internal) [12]. From industrial data, there are several types of sensor errors according to the source of uncertainty. Sensor error modes are classified into three types, i.e., delay, offset, and stuck. Delay errors refer to the delayed availability of sensor data, offset errors include sporadic offset, stochastic offset, and permanent offset including drift error, and stuck errors such as stuck at constant and stuck at zero refer to a series of measurements with inaccurate constant values. These error modes are mainly caused by deviation from real values, as well as the occurrence of oscillation. All errors can lead to critical, negative effects in NPPs, such as a malfunction of an autonomous safety system or a wrong alarm. 

Among the above possible error modes, the following two modes are selected here considering their influence on human error during diagnosis tasks: (1) drift error which is a time-correlated permanent offset error and (2) stuck at constant error. Accident diagnosis is mainly determined by checking the parameter trends or whether the parameters have exceeded their thresholds; as the drift and stuck at constant errors exhibit similar behaviors as error-free operation, they are selected here as the main target errors to be detected. Other error modes are excluded from the analysis because, for example, the stochastic offset and stuck at zero error modes can be easily known by operators because of their discrepancy from real trends.

**Figure 2:**
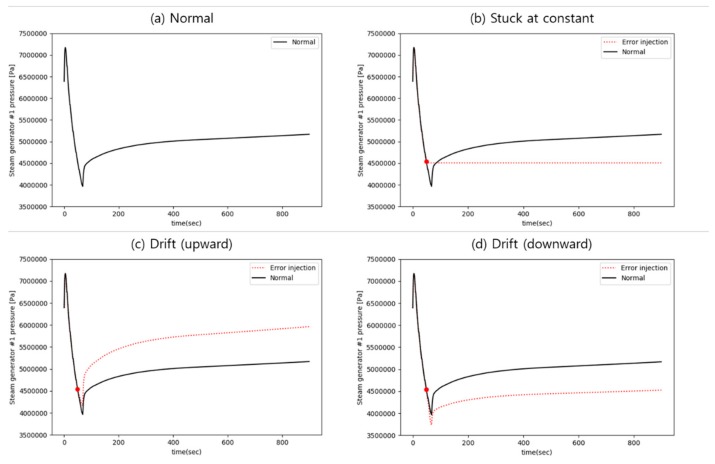
Examples of error injection. (**a**) Normal; (**b**) Stuck at constant; (**c**) Slow drift (upward); and (**d**) Slow drift (downward) sensor error during a loss of coolant accident.

For implementation of the selected error modes, a uniform method of error injection is applied to all sensors regardless of the type or environment of the sensor. First, the stuck at constant error is implemented by fixing the sensor value at the error injection point. Second, the drift error is divided into slow and rapid drift according to the slope of the change. Each drift is implemented with rates of change of two and five times from the real trends. In cases with no changes of the real values, each drift is implemented with 0.4% and 4% of the fixed values. Additionally, each drift is also split into upward and downward drift by the drift direction. In sum, the injected errors in this work are (1) stuck at constant, (2) slow upward drift, (3) slow downward drift, (4) rapid upward drift, and (5) rapid downward drift. Figure 2 plots examples of the injected error data against the normal parameter trend.

### 4.3. Data Preprocessing

For efficient training of the LSTM network, two data preprocessing steps are performed. The first is a Gaussian smoothing of the time series parameters. Parameter oscillation is typically observed in LOCA data, estimated to derive from the vaporization of coolant, and such oscillation can cause the false detection of sensor error. Therefore, a one-dimensional Gaussian smoothing filter is applied, in which the Gaussian distribution function used for smoothing is given by:

(3) $$G\left(x\right)=\frac{1}{\sigma \sqrt{2\pi }}\exp^{\frac{-x^{2}}{2\sigma^{2}}}$$

**Figure 3:**
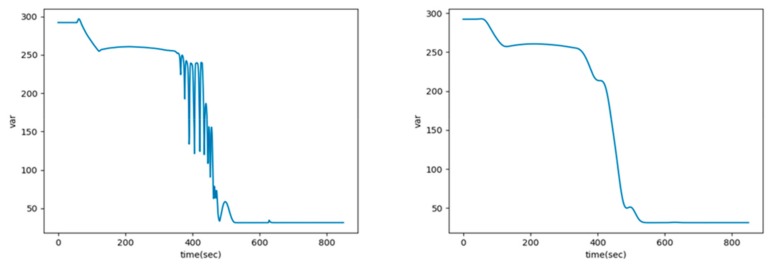
Example parameter oscillation (left) and smoothing result (right).

Following the value of the standard deviation σ, the width of the Gaussian distribution, or in other words the effect of the smoothing, is determined [39]. Gaussian smoothing with σ=100 is applied to the oscillating parameters. As shown in Figure 3, this step eliminates the oscillations while maintaining the parameter trend.

The second preprocessing step is a min-max normalization of all parameters. As seen in Table 1, various parameter scales are used for training the LSTM network. In order to reflect all plant parameters, normalization is needed. Minimum and maximum parameter values along the entire accident sequence are collected and used for the normalization as follows:

(4) $$x_{norm}=\frac{x-x_{min}}{x_{max}-x_{min}}.$$

### 4.4. Long-Short Term Memory Network

**Figure 4:**
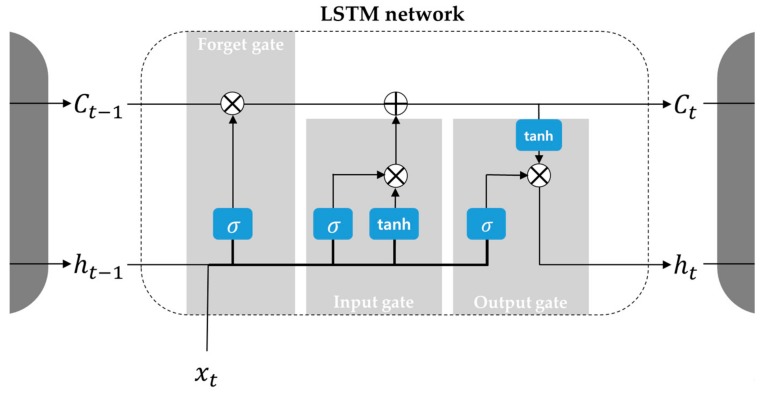
Long short-term memory (LSTM) network scheme.

Recurrent neural networks (RNNs) have special structural connections between nodes and each node has memory to process inputs from the present state as well as from connected nodes. This structure enables the system to consider the temporal context of data [40,41,42]. However, RNNs contain the vanishing gradient problem, meaning they cannot learn the long-term dependencies of the data [43]. To solve this, a particular RNN structure called the long-short term memory (LSTM) network was suggested [44]. The LSTM network has a hidden cell state that is controlled by three gate functions, i.e., input, forget, and output gate functions, as shown in Figure 4. The functions are computed by Equations (5) to (10) as below:

(5) $$i_{t}=\sigma \left(W_{i}[x_{t},h_{t-1}]+b_{i}\right),$$

(6) $$f_{t}=\sigma \left(W_{f}[x_{t},h_{t-1}]+b_{f}\right),$$

(7) $$o_{t}=\sigma \left(x_{t}U^{o}+h_{t-1}W^{o}\right),$$

(8) $${\tilde{C}}_{t}=tan\;h\left(W_{C}[x_{t},h_{t-1}]+b_{C}\right),$$

(9) $$C_{t}=\sigma \left(f_{t}*C_{t-1}+i_{t}*{\tilde{C}}_{t}\right),$$

(10) $$h_{t}=tan\;h\left(C_{t}\right)*o_{t},$$

where 𝑊 is the weight matrix of each gate and *b* is the bias. At the forget gate ($f_{t}$), information is kept or discarded according to the previous output and the present value. The input gate vector ($i_{t}$) determines the updates to the cell state vector ($C_{t}$) with candidate values (${\tilde{C}}_{t}$). The output vector of the current block ($h_{t})$ is determined by an activation vector ($o_{t}$) and the cell state vector. With such a setup, short-term characteristics are reflected in the output vector, and long-term dependencies are reflected in the cell state vector. In our system, the LSTM network consists of an input layer, two hidden layers, and an output layer. The input layer receives time series data as input *x*, which is multiple sensor data in our model. Following the time steps, information transfer occurs to train the contextual information with the three gate functions. The network generates output at every time step. The estimated output, which is the consistency index in our model, is compared to the real consistency index, which is the correct answer of the output. To reflect estimation error, the network back propagates (in the reverse direction of time) to update the weights for more accurate estimation. Training and validation with related datasets are done iteratively. Ultimately, the LSTM model gains the ability to generate consistency index values for times series input data.

In terms of the model selection, a pilot study was conducted that also tested a deep multilayer perceptron model [45] and a convolutional neural network model [46]; both neural network models were excluded from the present analysis due to poor performance as compared with the LSTM network. Likewise, Bae et al. (2019) recently compared multilayer perceptron and LSTM networks for plant parameter prediction in nuclear emergency situations and showed much more accurate results with the LSTM network [47]. 

## 5. Applications

### 5.1. Data Extraction

Since NPP emergency situations occur at rare intervals, related process parameter data from plant experience is insufficient. Therefore, a CNS developed by the Korea Atomic Energy Research Institute was employed to generate accident data. A CNS is a compact-scale NPP simulator implementing a three-loop pressurized water reactor from Westinghouse. It is based on the SMABRE thermal-hydraulic system code that was developed for analyzing small-break loss of coolant accidents or leak transient. The SMABRE code is a one-dimensional model with simplified assumptions and experimental data. It cannot reflect all possible phenomena or events in real emergency accident situations. Nonetheless, it has advantages of good accessibility and fast calculation for the production of large amounts of accident data [48,49,50,51].

Among the emergency situations covered by the ORPs, the following four accidents were selected: LOCA, SGTR, ESDE, and LOAF accidents. Reactor trips with no specific symptoms or accidents from power loss were excluded from analysis due to a lack of distinguishable symptoms in the CNS. After the related malfunctions of the selected accidents were injected to normal CNS operation, a reactor trip occurred by an autonomous signal. From the reactor trip, time series data of certain plant parameters were collected for 15 min, corresponding to the recommended time limit for accident diagnosis [34]. Twenty-one plant parameters were selected based on the diagnosis procedure and their importance to accident estimation. The time interval of data collection was 1 s, and thus 900 time points were collected per dataset. For data variation, nine break sizes and three break locations were considered in creating LOCA, SGTR, and ESDE accident data. 

For the purpose of validating the proposed system, the following target sensors were chosen considering their importance in diagnosis: sensors monitoring pressurizer pressure; containment radiation; secondary system radiation; steam generator #1, #2, #3 pressure and water level; reactor vessel water level, cold-leg #1 temperature, hot-leg #1 temperature, and core outlet temperature. Pressurizer pressure is a parameter affected by all types of accidents, and, accordingly, it is used by operators for a rough diagnosis of an accident; the other 13 parameters are important to diagnose or estimate LOCA, SGTR, ESDE, and LOAF. The injected errors had six time points at the early phase of reactor trip with 60 s intervals for training and validation datasets. The test set also had the same time interval with three different time points. For the training of each single failure of the target sensors, 7488 training sets, 2223 validation sets, and 3159 test sets were used.

### 5.2. Application Results

**Figure 5:**
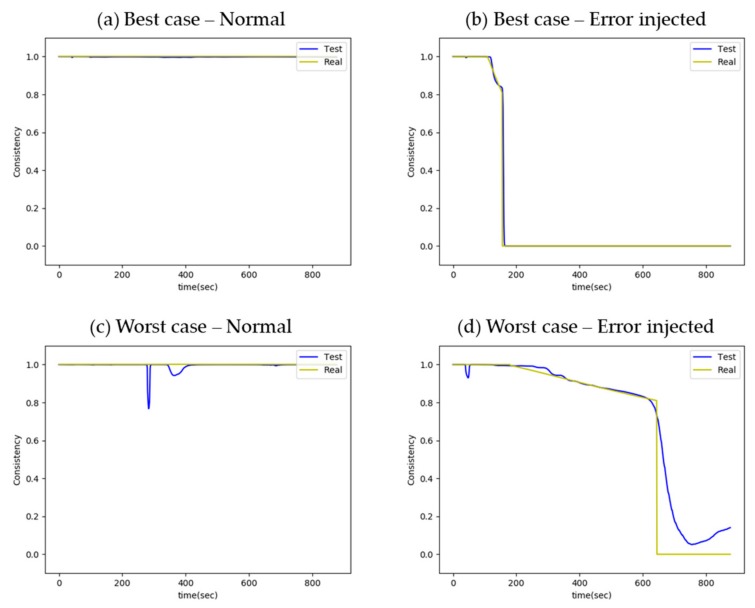
(**a**–**d**) Consistency trends of the best and worst cases from normal and error-injected tests.

Examples of the consistency trend output are shown in Figure 5. The yellow lines depict the real consistency trends of the target sensor, obtained by labeling both normal and error-injected raw data with consistency values, and the blue lines show the estimated consistency values of the target sensor from the LSTM network test output. In the best case with a normal sensor, as shown in Figure 5a the estimated result clearly shows the soundness of the sensor. The estimated consistency also effectively follows the drop of the real consistency value, as shown in Figure 5b, with detection of the injected error. However, the estimated consistency, as shown in Figure 5c, has a dip in the early phase of the reactor trip; this is because drastic changes of all parameters, as well as the error injection are all located at the front of the accident sequence. In the worst case with error-injected data, as shown in Figure 5d, consistency decreases slowly. As each parameter has various rates of change and trends, the consistency trends also vary. Nevertheless, in this case, the estimation drops in the latter part, following the real consistency value.

#### 5.2.1. Error Criteria

Test error datasets in which the real consistency index label did not drop to zero were excluded from results analysis because they are not perceived as faulty sensors in our system. Likewise, for all test data, sensor success and fault criteria need to be identified. Setting higher criteria results in the quick detection of sensor error; however, this can be accompanied by increases in the false detection of sensor error. Table 2 lists the number of datasets in terms of the minimum consistency index location from trained LSTM test outputs. The results clearly discriminate the consistency between normal and error cases. In the normal case with no sensor errors, 97.49% of the data maintained an index value of over 0.9, while the other 0.79% and 1.72% of the data were over 0.7 and 0.8 during the accident sequence. In the error case, 72.77% of error data were distributed under the 0.1 index, 27.10% between 0.1 and 0.2, and 0.13% between 0.2 and 0.3. Error criteria should be decided based on the ability to distinguish between normal and error states of the sensor; therefore, according to the results, the error criteria should be set between 0.3 and 0.7. In accordance with the steady output of the normal case, a higher error criterion of 0.7 was chosen for sensor error detection. 

#### 5.2.2. Error Detection Time Analysis

The average time to reach all consistency indexes in the error test data was collected, as shown in Table 3. The average time for all error data was 116.11 s. The decrease of consistency accelerates after reaching the consistency value of 0.8.

Table 4 and Table 5 contain the average times for the sensor error detection in the present application. Note that in all the normal cases, the system succeeded to produce output with the true value; the results from normal data can, therefore, be classified as “success” or “failure” by whether the index value was maintained above the error criteria over all time steps or not, and thus all the normal cases were classified as “success”. Among the stuck-at-constant error cases, some test data had no meaningful results (as marked with dashes) because the real values also remained constant during the accident sequences. The detection time varied according to the type of accident, error modes, and target sensors because the trend or features of the parameters were completely different in each accident. Rapid drifts were detected earlier than the other errors, but the stuck and slow drift error showed unstable results. Errors in containment radiation and secondary radiation accidents were detected within a relatively short time as compared with the other errors. This result came from the related simple parameter trends along with the characteristic of being clearly distinguished from other parameters.

The results of Table 2 present the performance of the model to distinguish between normal and error data for all test sets. As previously mentioned, the error criteria value was determined based on the observed uncertainty of these results; using this criterion (0.7), the system demonstrated successful performance. However, accumulation of training data and incorporating more accident types can increase the degree of peak points that can lead to false detection. The possibility for false detection of sensor error is identified as a potential negative effect of the error detection model. If a false case is observed, more logics to determine the sensor error can be added to prevent recurrence. Possible methods for this include the application of a cumulative function or a downward adjustment of the error criteria.

## 6. Conclusions

A framework for a sensor error detection system designed for nuclear power plant emergency situations using a machine learning model has been presented. The performance of the system was tested with emergency situation simulation data with a single sensor failure. The proposed system is differentiated from previous approaches, such as OLM techniques that are based on signal reconstruction, as the developed system generates consistency index values to express the soundness of the sensors, thereby allowing the reliability of the sensors to be quantified during typical emergency sequences. To handle the complex combinations of systems in NPPs, a LSTM network was applied with successful results obtained. Ultimately, this study shows the applicability of the sensor error detection system to NPP emergency situations.

Recently in the nuclear field, data-driven methods to support accident response are being actively studied with the advancement of computing power. Such methods include the prediction of plant parameters, the diagnosis of accidents, and autonomous operation during an accident [52,53,54]. The monitoring of sensor soundness can support the practical application of these incoming technologies. 

In this study, the sensor error modes that can result in possible human error were selected and artificially implemented as simulation data. As mentioned in Section 4.1, however, there are various types of sensor error depending on the sensor type and cause. For application of the system to a real plant, some future works need to be performed. First, the sensor error detection system should cover all possible errors, requiring the training and testing of more error modes. Second, a comparative evaluation of different machine learning models should be performed to determine whether more accurate and quick detection of sensor error is possible. Third, operator action and simultaneous sensor errors should also be considered.

## Figures and Tables

**Table 1:** Selected plant parameters from the compact nuclear simulator.

	Plant Parameter (Units)
1	PRESSURIZER LEVEL (m)
2	REACTOR VESSEL WATER LEVEL (m)
3	CONTAINMENT RADIATION. (mRem/hr)
4	COLD-LEG #1 TEMPERATURE (°C)
5	HOT-LEG #1 TEMPERATURE (°C)
6	CORE OUTLET TEMPERATURE. (°C)
7	STEAM GENERATOR #1 LEVEL, WIDE RANGE (m)
8	STEAM GENERATOR #2 LEVEL, WIDE RANGE (m)
9	STEAM GENERATOR #3 LEVEL, WIDE RANGE (m)
10	SECONDARY SYSTEM RADIATION (mRem/hr)
11	STEAM GENERATOR #1 PRESSURE (Pa)
12	STEAM GENERATOR #2 PRESSURE (Pa)
13	STEAM GENERATOR #3 PRESSURE (Pa)
14	PRESSURIZER PRESSURE (Pa)
15	FEED WATER LINE 1 FLOW (kg/sec)
16	FEED WATER LINE 2 FLOW (kg/sec)
17	FEED WATER LINE 3 FLOW (kg/sec)
18	CONTAINMENT SUMP WATER LEVEL (m)
19	STEAM LINE 1 FLOW (kg/sec)
20	STEAM LINE 2 FLOW (kg/sec)
21	STEAM LINE 3 FLOW (kg/sec)

**Table 2:** Location of minimum consistency index.

**C Index**	<0.1	0.1–0.2	0.2–0.3	0.3–0.7	0.7–0.8	0.8–0.9	>0.9	Total
**Normal**	0	0	0	0	17(0.79%)	37(1.72%)	2098(97.49%)	2152
**Error**	6751(72.77%)	2514(27.10%)	12(0.13%)	0	0	0	0	9277

**Table 3:** Average time to reach the *C* index in the error test data.

**C index**	0.9	0.8	0.7	0.6	0.5	0.4	0.3	0.2	0.1	0
**Detection Time (s)**	60.20	98.52	116.11	130.13	132.14	134.02	135.58	137.55	140.01	670.31

**Table 4:** Average sensor error detection time (1) (units, sec).

Accident	Error Mode	Pressurizer Pressure	Containment Radiation	Secondary Radiation	SG #1 Pressure	SG #1 Level	SG #2 Pressure	SG #2 Level
LOCA	Normal	success	success	success	success	success	success	success
	Stuck	71.56	16.26	—	218.02	15.17	236.11	138.85
	Slow drift	69.89	41.72	11.15	85.03	21.16	195.33	14.26
	Rapid drift	53.45	23.63	7.93	56.24	13.73	110.09	8.83
SGTR	Normal	success	success	success	success	success	success	success
	Stuck	46.80	—	206.35	58.67	60.69	15.36	91.83
	Slow drift	45.64	6.70	151.74	112.17	18.45	218.41	19.24
	Rapid drift	34.88	4.63	125.18	86.46	13.13	121.22	11.40
ESDE	Normal	success	success	success	success	success	success	success
	Stuck	99.71	—	—	173.48	60.17	85.40	118.27
	Slow drift	81.35	5.98	10.34	90.69	81.36	103.10	45.85
	Rapid drift	52.59	4.17	7.98	54.59	39.78	71.03	36.37
LOAF	Normal	success	success	success	success	success	success	success
	Stuck	24.33	—	—	63.00	13.00	112.00	9.50
	Slow drift	17.83	4.00	11.50	56.00	9.83	119.50	11.92
	Rapid drift	16.17	2.50	8.50	45.17	5.00	80.00	5.58

**Table 5:** Average sensor error detection time (2) (units, sec).

Accident	Error Mode	SG #3 Pressure	SG #3 Level	Reactor Vessel Water Level	Cold-Leg #1 Temperature	Hot-Leg #1 Temperature	Outlet Temperature
LOCA	Normal	success	success	success	success	success	success
	Stuck	220.25	15.39	140.09	343.00	—	338.00
	Slow drift	82.35	21.72	129.30	342.67	334.41	355.51
	Rapid drift	15.29	13.79	92.38	128.00	134.72	135.52
SGTR	Normal	success	success	success	success	success	success
	Stuck	17.27	116.30	246.11	—	—	319.50
	Slow drift	125.59	19.54	21.63	311.11	294.01	282.58
	Rapid drift	64.83	14.07	14.06	168.56	175.07	175.45
ESDE	Normal	success	success	success	success	success	success
	Stuck	77.38	149.75	10.00	161.41	320.50	192.28
	Slow drift	111.77	64.55	66.46	221.59	286.41	246.61
	Rapid drift	63.98	40.41	3.37	97.10	178.28	117.52
LOAF	Normal	success	success	success	success	success	success
	Stuck	110.00	12.67	—	279.00	296.00	296.50
	Slow drift	117.50	12.33	66.67	179.33	138.33	139.67
	Rapid drift	79.00	7.00	3.67	77.33	85.00	83.67
